# The third dose of mRNA SARS-CoV-2 vaccines enhances the spike-specific antibody and memory B cell response in myelofibrosis patients

**DOI:** 10.3389/fimmu.2022.1017863

**Published:** 2022-09-29

**Authors:** Fabio Fiorino, Annalisa Ciabattini, Anna Sicuranza, Gabiria Pastore, Adele Santoni, Martina Simoncelli, Jacopo Polvere, Sara Galimberti, Claudia Baratè, Vincenzo Sammartano, Francesca Montagnani, Monica Bocchia, Donata Medaglini

**Affiliations:** ^1^ Laboratory of Molecular Microbiology and Biotechnology, Department of Medical Biotechnologies, University of Siena, Siena, Italy; ^2^ Hematology Unit, Department of Medical Science, Surgery and Neuroscience, Azienda Ospedaliero Universitaria Senese, University of Siena, Siena, Italy; ^3^ Section of Hematology, Department of Clinical and Experimental Medicine, University of Pisa, Pisa, Italy; ^4^ Department of Medical Biotechnologies, University of Siena, Siena, Italy; ^5^ Department of Medical Sciences, Infectious and Tropical Diseases Unit, Azienda Ospedaliero Universitaria Senese, University Hospital of Siena, Siena, Italy

**Keywords:** mRNA SARS-CoV-2 vaccination, COVID-19, myelofibrosis, third booster dose, ruxolitinib, antibody response, B-cell response

## Abstract

Vaccination against SARS-CoV-2 using mRNA-based vaccines has been highly recommended for fragile subjects, including myelofibrosis patients (MF). Available data on the immune responsiveness of MF patients to mRNA SARS-CoV-2 vaccination, and the impact of the therapy with the JAK inhibitor ruxolitinib, are still fragmented. Here, we profile the spike-specific IgG and memory B-cell response in MF patients, treated or not with ruxolitinib, after the second and the third dose of SARS-CoV-2 BNT162b2 (BioNTech) and mRNA-1273 (Moderna) vaccines. Plasma and peripheral blood mononuclear cells samples were collected before vaccination, post the second and the third doses and tested for spike-specific antibodies, ACE2/RBD antibody inhibition binding activity and spike-specific B cells. The third vaccine dose significantly increased the spike-specific IgG titers in both ruxolitinib-treated and untreated patients, and strongly enhanced the percentage of subjects with antibodies capable of *in vitro* blocking ACE2/RBD interaction, from 50% up to 80%. While a very low frequency of spike-specific B cells was measured in blood 7 days after the second vaccination dose, a strong and significant increase was elicited by the third dose administration, generating a B cell response similar to the one detected in healthy controls. Despite the overall positive impact of the third dose in MF patients, two patients that were under active concomitant immunosuppressive treatment at the time of vaccination, and a patient that received lymphodepleting therapies in the past, remained low responders. The third mRNA vaccine dose strongly increases the SARS-CoV-2 specific humoral and B cell responses in MF patients, promoting a reactivation of the immune response similar to the one observed in healthy controls.

## Introduction

Patients with hematologic malignancies are at an increased risk of Severe Acute Respiratory Syndrome Coronavirus 2 (SARS-CoV-2) infection and report highest disease severity and death rates compared to the general population (1–5). Booster doses of SARS-CoV-2 vaccine have been highly recommended and prioritized in fragile categories including patients with myelofibrosis (MF), a clonal hematopoiesis stem cell disorder belonging to the Philadelphia-negative myeloproliferative neoplasms (MPN). MF is characterized by bone marrow fibrosis, progressive cytopenia, and extramedullary hematopoiesis, with complications as anemia, opportunistic infections, and ultimately progression to leukemia in a fraction of patients. MF patients may receive clinical benefits from ruxolitinib, the first approved JAK1/JAK2 inhibitor (6) that deeply reduces inflammatory cytokine production and impairs to some extent cellular immune responses (7). Indeed, ruxolitinib has been successfully employed in attenuating the cytokine storm responsible of fatal acute respiratory distress syndrome in severe COVID-19 disease, as reported by several studies (8, 9). On the other hand, the interruption of ruxolitinib treatment in SARS-CoV-2 infected MF patients has been followed by an increase in death rate, probably due to the cytokine rebound subsequent to the drug suspension (10).

We previously demonstrated a slower kinetic of antibody response in MF patients vaccinated with two doses of mRNA SARS-CoV-2 vaccine compared to healthy subjects, and a reduced ACE2/RBD inhibition binding capacity of plasma antibodies, especially in concomitance with ruxolitinib treatment (11). The reduced response in MF patients, particularly if under ruxolitinib treatment, was also confirmed by Cattaneo et al. (12). The immune response kinetic observed in these subjects, showing a reduced capability of their immune system to rapidly react to vaccination, strongly suggested the need of booster vaccine dose (11), that was indeed recommended by the Centers for Disease Control and Prevention (CDC) for “fragile” subjects. On the matter we indeed recently demonstrated how the third dose of mRNA-1273 improved SARS-CoV-2 immunity in patients with hematologic malignancies treated with autologous and allogeneic hematopoietic cell transplantation, which exhibit a low response to the first cycle of vaccination (13).

Existing data on small groups of MF subjects are focused largely on antibody levels and their ability to neutralize the virus (14–17). However, besides the antibody immune response, it’s of critical importance to profile the induction and persistence of antigen-specific cellular immune responses. In particular, memory B cells induced by vaccination are capable of reactivation upon pathogen encounter, with secretion of novel wave of antibodies (18). For this reason, the assessment of spike-specific B cell response is crucial to characterize the long-term persistence of effective immune responses even beyond the decline of circulating antibodies (19). Our studies on immune responsiveness in healthy subjects vaccinated with nanoparticles-based mRNA formulations (20) clearly show the persistence of circulating spike-specific antibodies and immune memory B cells six months after the first cycle of vaccination with BNT162b2 (21), and we have followed up their persistence in blood up to 9 months (Ciabattini et al., manuscript in preparation).

In the present work, we longitudinally profiled the immune response after the second and the third booster dose of mRNA vaccines (Spikevax mRNA-1273 or Comirnaty BNT162b2) in a cohort of MF patients referring to the Hematology Unit of the Siena University Hospital. The analysis focused on the anti-spike antibody response, their ACE2/RBD binding inhibition activity, and the characterization of the spike-specific B cell response, also evaluating the potential impact of ruxolitinib therapy on the vaccine immune responsiveness.

## Material and methods

### Study design

**Figure 1:**
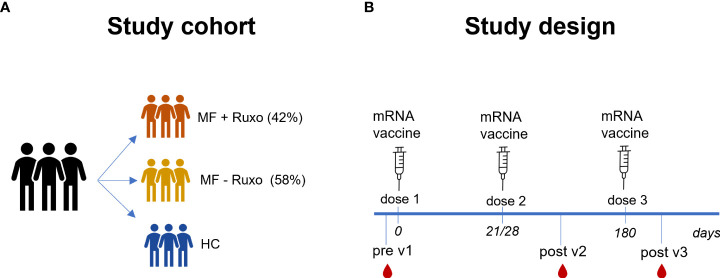
Schematic representation of the study cohort and design. **(A)** Patients with myelofibrosis (MF), treated or not with ruxolitinib (Ruxo), and healthy controls (HC) were immunized with three doses of mRNA vaccines anti SARS-CoV-2. **(B)** Antibody and B-cell responses were evaluated at the baseline and after the second and the third vaccination dose.

Nineteen MF patients and healthy volunteers (HC) vaccinated with mRNA SARS-CoV-2 vaccine (Spikevax mRNA-1273 or Comirnaty BNT162b2), were enrolled in the study (**Table 1**). Patients with MF were treated with ruxolitinib (8/19; MF+Ruxo) or received hydroxyurea (HU)/supportive therapy only (11/19; MF-Ruxo, **Figure 1A**). mRNA vaccines were administered at day 0, 21/28 (BNT162b2 and mRNA-1273, respectively) and 6 months after the second dose. Plasma and peripheral blood mononuclear cells (PBMCs) samples were collected at baseline (hereafter indicated as pre v1), 7-30 days after the second vaccine dose (post v2), and 15 days after the third vaccine dose (post v3; **Figure 1B**) and tested for spike-specific antibodies, ACE2/RBD inhibition binding activity and spike-specific B cells.

**Table 1:** Demographic characteristics of myelofibrosis (MF) patients and healthy controls.

Subject characteristics	MF cohort			Healthy controls
	with ruxolitinib (*n* = 8)	without ruxolitinib (*n* = 11)	Total (n=19)	Total (20)
Age^a^, mean years (range)	72 (47—88)	72 (44—77)	72 (45—89)	522 (32—70)
Sex Male Female	2 (25%) 6 (75%)	6 (54.6%) 5 (45.4%)	8 (42%) 11 (58%)	11 (55%) 9 (45%)

a Age at the first vaccine dose.

The study was performed in compliance with all relevant ethical regulations, and the protocol was approved by the local Ethical Committee for Clinical experimentation of Regione Toscana Area Vasta Sud Est (CEAVSE), with the protocol code 19479 PATOVAC_COV v1.0, approved on March 15^th^ 2021, and the protocol code 18869 IMMUNO_COV v1.0, approved on Novembrer 18^th^ 2020. All subjects provided written informed consent before participation in the study. Inclusion criteria were confirmed diagnosis of MF, age ≥ 18 years, adhesion to the COVID-19 vaccination campaign; exclusion criteria included pregnancy, withdrawal of consent or refusal to participate, clinical problems for collecting additional blood samples beyond the amount required for routine care, participation to other clinical trials.

Study participants without any sign or syntoms of SARS-CoV-2 infection in anamnesis were recruited at the Hematology Unit, Azienda Ospedaliero Universitaria Senese (Siena, Italy). Clinical data collection and management were carried out using the software REDCap (Research Electronic Data Capture, Vanderbilt University).

### PBMCs isolation

Venous blood samples were collected in a heparin coated blood tube (BD Vacutainer) at pre v1, post v2 and post v3. PBMCs were isolated by density-gradient sedimentation, using Ficoll-Paque (Lymphoprep, Voden Medical Instrument, Meda, Italy). Isolated PBMC were then cryopreserved in cell recovery medium [10% DMSO (Thermo Fisher Scientific) and 90% heat inactivated fetal bovine serum (Sigma Aldrich)] and stored in liquid nitrogen until used. Plasma samples were stored at −80°C.

### ELISA

Maxisorp microtiter plates (Nunc, Denmark) were coated with recombinant SARS-CoV-2 full spike protein (S1 + S2 ECD, Sino Biological) with 50 μL per well of 1 μg/mL protein solution in PBS (Sigma-Aldrich, St. Louis, MO, USA), and left overnight at 4°C. Plates were blocked at room temperature (RT) for 1 h with 200 μL of 5% skimmed milk powder (AppliChem, Darmstadt, Germany), 0.05% Tween 20, 1 × PBS. Heat-inactivated plasma samples were added and titrated in two-fold dilution in duplicate in 3% skimmed milk powder, 0.05% Tween 20 (Sigma-Aldrich), 1 × PBS (diluent buffer) and incubated 1 h at RT. Anti-human horseradish peroxidase (HRP)-conjugated antibody for IgG (diluted 1:6000; Southern Biotechnology, Birmingham, AL, USA) was added in diluent buffer for 1 h at RT. Plates were developed with 3,3′,5,5′-Tetramethylbenzidine (TMB) substrate (Thermo Fisher Scientific, Waltham, MA, USA) for 10 min at RT, followed by addition of 1M stop solution. Absorbance at 450 nm was measured on Multiskan FC Microplate Photometer (Thermo Fisher Scientific). WHO international positive control (plasma from vaccinated donor; NIBSC) and negative control (plasma from unvaccinated donor, NIBSC) were added in duplicate to each plate as internal controls for assay reproducibility. Antibody end point titers were expressed as the reciprocal of the sample dilution, reporting double the background OD value. A titre > 640 was considered as positive.

### ACE2/RBD binding inhibition assay

ACE2/RBD binding inhibition was tested with the SARS-CoV-2 surrogate virus neutralization test (sVNT) kit (cPass™ SARS-CoV-2 Neutralization Antibody Detection Kit, Genscript, Piscataway, NJ, USA) according to the manufacturer protocol. Plasma samples, positive and negative controls were diluted 1:10 in dilution buffer, mixed 1:1 with HRP-RBD buffer and incubated for 30 min at 37°C. An amount of 100 µL of each mixture was added to each well of an ACE2-coated 96-well flat-bottom plate and incubated for 15 min at 37°C. After washing steps, the volume of 100 µL of TMB solution was added to each well and the plate was developed for 15 min at RT. The reaction was quenched by adding 50 µL of the stop solution to each well, and the OD at 450 nm was instantly read with Multiskan FC Microplate Photometer (Thermo Fisher Scientific). Results of the ACE2/RBD inhibition assay were expressed as follows: percentage inhibition = (1 − sample OD value/negative control OD value) × 100. Inhibition values ≥30% was considered as positive results, and values <30% as negative results, as established by Tan at al (22) and indicated by the manufacturer.

### Multiparametric flow cytometry

A 7-color panel was developed to phenotype B-cell populations and identify SARS-CoV-2-specific B cells among PBMC by flow cytometry. The biotinylated spike proteins were tetramerized with fluorescently labeled streptavidin (SA) as follows: Spike S1+S2 ECD-His recombinant biotinylated-protein (Sino Biological) with SA-R-Phycoerythrin (PE), RBD recombinant biotinylated-protein (BioLegend) with SA-Allophycocyanin (APC). Two million PBMCs were incubated with BD human FC block (BD Biosciences) for 10 min at RT, and stained for 30 min at 4°C with the following antibody-fluorochrome panel: CD3-BV650 (clone SK7); CD19-BUV395 (clone SJ25C1), IgD-BV711 (clone IA6-2), CD20-APCH7 (clone 2H7), CD27-BV786 (clone M-T271), CD38-BUV737 (clone HB7, all from Becton Dickinson). Following surface staining, cells were washed once with PBS and labeled with Live/Dead Zombie according to the manufacturer instruction (Thermofisher). Cells were washed once with PBS, resuspended in 100 µL BD fixation solution (BD Biosciences) and incubated at 4°C for 15 min in the dark. All antibodies were titrated for optimal dilution. About 1-2 × 10^6^ cells were acquired and stored for each sample with SO LSRFortessa X20 flow cytometer (BD Biosciences). Data analysis was performed using FlowJo v10 (TreeStar, USA).

### Statistical analysis

Kruskal-Wallis test, followed by Dunn’s post test for multiple comparisons, was used for assessing statistical differences between different groups for each time point. Unpaired Mann-Whitney test was used to assess statistical difference for each group at different time point. A p-value ≤ 0.05 was considered significant. Analyses were performed using GraphPad Prism v9 (GraphPad Software, San Diego, CA, USA).

## Results

Nineteen MF patients were enrolled in the study and profiled for the spike-specific antibody and memory B cell responses following administration of SARS-CoV-2 mRNA vaccines. All subjects received Spikevax mRNA-1273 for the first vaccination cycle, and Spikevax mRNA-1273 (17 patients) or Comirnaty-BNT16b2b (2 patients) for the third dose. The median age of this cohort was 72 years (range 44-88 years), 8 were male (42%) and 11 were female (58%). A cohort of 20 age and sex-matching healthy controls (HC) was included in the study (**Table 1**). At the time of the first vaccination 8 out of 19 MF patients (42%) were treated with ruxolitinib while 11 (58%) were on supportive care and/or HU only. Complete clinical characteristics of MF patients are summarized in **Table 2**. Of note, a patient (#583) in therapy with ruxolitinib had a concomitant treatment with ibrutinib for a diagnosis of chronic lymphocytic leukemia (**Table S1**). A schematic representation of the study design is reported in **Figure 1**.

**Table 2:** Clinical characteristics of MF patients before the third dose of mRNA vaccine anti SARS-CoV-2.

Clinical characteristics	MF Whole cohort (*n* = 19)	MF with ruxolitinib (*n* = 8)	MF without ruxolitinib (*n* = 11)
BMI (Kg/m^2^), median (range)	21,3 (17,5–26,2)	21,2 (18,5–25,1)	21,7 (17,5–26,2)
Disease Primary MF Post–PV Post–ET	12 (63.2%) 5 (26.3%) 2 (10.5%)	3 (37.5%) 4 (50%) 1 (12.5%)	9 (82%) 1 (9%) 1 (9%)
IPSS SCORE LOW INT–1 INT–2 HIGH	4 (21%) 8 (42.1%) 4(21.1%) 3 (15.8%)	1 (12.5%) 4 (50%) 1 (12.5%) 2 (25%)	3 (27%) 4 (37%) 3 (27%) 1 (9%)
Driver mutation JAK2 CALR MPL Triple negative	13 (69.1%) 5 (21.4%) 0 (0%) 1 (7.1%)	8 (100%) 0 (0%) 0 (0%) 0 (0%)	5 (45.5%) 5 (45.5%) 0 (0%) 1 (9%)
Exposition to ruxolitinib, months median (range)		20.5 (6–42)	
Spleen below costal margin, cm median (range)	6.5 (0–22)	12 (0–22)	5 (0–13)
Hemoglobin g/dL, median (range)	13 (8.6–14.8)	13 (9.5–13.4)	13 (8.6–14.8)
Platelets × 10^3^/µL, median (range)	395 (27–781)	183 (27–680)	458 (60–781)
WBC × 10^3^/µL, median (range)	9.45 (3.6–33.9)	16.3 (3.6–33.9)	9.45 (4.3–13.7)
Lymphocytes × 10^3^/µL, median (range)	1.76 (0.93–6.02)	1.44 (1.07–6.02)	1.8 (0.93–3.79)
Total protein g/dL, median (range)	6.7 (5.7–7.9)	6.4 (6–7.9)	6.8 (5.7–7.1)
γ-Globulins (%), median (range)	15.2 (7.3–23.3)	15.2 (14.1–23.3)	14.6 (7.3–19.9)
LDH U/L, median (range)	422 (191–1384)	391 (235–838)	443 (191–1384)

BMI, Body Mass Index; PMF, Primary Myelofibrosis; PV, Polycythemia vera; ET, Essential Thrombocythemia; WBC, White Blood Count (x10^3^/µL); LDH, Lactate Dehydrogenase (U/L).

### Spike-specific antibody response and ACE2/RBD inhibition binding activity

**Figure 2:**
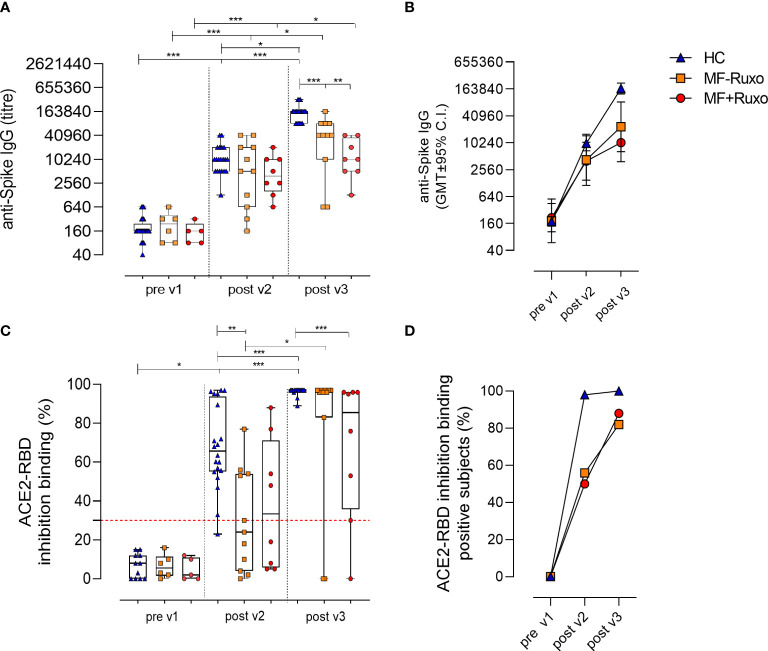
Humoral immune response against SARS-CoV-2 in patients with myelofibrosis (MF) after the second and the third dose of mRNA vaccine. **(A)** Spike-specific IgG titres were assessed by ELISA in plasma of patients with MF, treated or not with ruxolitinib (Ruxo), and healthy controls (HC), at baseline (pre v1), and after two and three vaccine doses. Antibody end point titres were expressed as the reciprocal of the sample dilution reporting double the background OD value. **(B)** Time course of spike-specific IgG in plasma collected at the baseline (pre v1) and after two (post v2) and three (post v3) doses of mRNA SARS-CoV-2 vaccine. **(C)** SARS-CoV-2 surrogate virus neutralization assay was tested at baseline (pre v1), post v2 and post v3 in plasma by evaluating ACE2/RBD binding inhibition activity. Results are expressed as ACE2/RBD binding inhibition percentage with box and whiskers diagram showing all subject values. Inhibition values ≥30% are considered a positive result according to the manufacturer indication. **(D)** Percentage of subjects developing ACE2/RBD inhibition binding positive values (≥30%) in different groups. Kruskal-Wallis test, followed by Dunn’s post test for multiple comparisons, was used for assessing statistical differences between groups. Unpaired Mann-Whitney test was used to assess statistical difference for each group at different time point. *P ≤ 0.05; **P ≤ 0.01; ***P ≤ 0.001.

The induction and persistence of circulating spike-specific IgG was monitored following the second and the third vaccine dose (**Figures 2A, B**). Plasma levels of anti-spike IgG, tested 30 days after the second vaccine dose in MF patients, were not significantly different compared to HC regardless of ruxolitinib treatment, with GMT of 4238 in MF-Ruxo (95% CI 1134 to 15843; range 160-40960), 3948 in MF+Ruxo (95% CI 1487 to 10482; range 640-20480) and 9891 in HC (95% CI 6721 to 14556; range 1280-40960; **Figure 2A**).

The third vaccine dose significantly boosted the anti-spike IgG response, reaching antibody levels with GMT of 23231 in MF-Ruxo (95% CI 6455 to 83608; range 640-163840; P<0.01), 10240 in MF+Ruxo (95% CI 3845 to 27271; range 1280-40960) and 147661 in HC (95% CI 118783 to 183559; range 81920-327680; P<0.001, **Figure 2A**). However, the anti-spike IgG titers elicited were significantly lower in MF patients, regardless of ruxolitinib treatment, compared to HCs (P<0.01, **Figures 2A, B**). A significant increase of anti-spike IgG was observed for all groups after the second and the third vaccine dose compared to the baseline (P<0.05, **Figure 2A**). Based on the threshold indicated by the WHO for Middle East respiratory syndrome coronavirus and confirmed for SARS-CoV-2 (23), indicating antibody seroconversion with an increase > 4 respect to the baseline, the number of MF subjects showing a seroconversion of anti-spike IgG was 15/19 (79%) after the second vaccine dose (GMT of 3950, 95% CI 1842 to 8474; range 160-40960) and 17/19 (89%) after the third dose (GMT of 17199, 95% CI 7804 to 37903; range 640-163840).

Plasma samples collected before vaccination and after the second and the third vaccine dose were tested for their ability of blocking the interaction between RBD and ACE-2 receptor, employing a surrogate virus neutralization assay (22). Among MF patients, antibodies with a positive ACE2-RBD inhibition activity [value ≥30%, as reported in (22)] were observed in 45.5% of subjects without treatment (5/11; P≤0.01 *vs* HCs) and in 50% of Ruxo-treated MF patients (4/8) after the second vaccine dose (**Figure 2D**). After the third dose, ACE2-RBD inhibition activity was observed in 88% (7/8) of Ruxo-treated and 82% (9/11) of untreated MF patients, with a significant increase compared to post v2 (**Figure 2D**). Healthy subjects positive for ACE2-RBD inhibition were 98% post v2 (19/20) and 100% post v3 (20/20).

Taken together, the booster dose administered to MF patients significantly increased the anti-spike IgG response compared to levels observed after the second dose, regardless of the ruxolitinib treatment, and strongly raised the percentage of patients with antibodies capable of inhibiting the ACE2/RBD binding (P<0.05). Compared to HC, the spike-specific antibody response in MF patients was however lower (P<0.05, **Figures 2C, D**).

### Spike-specific B cells generation

**Figure 3:**
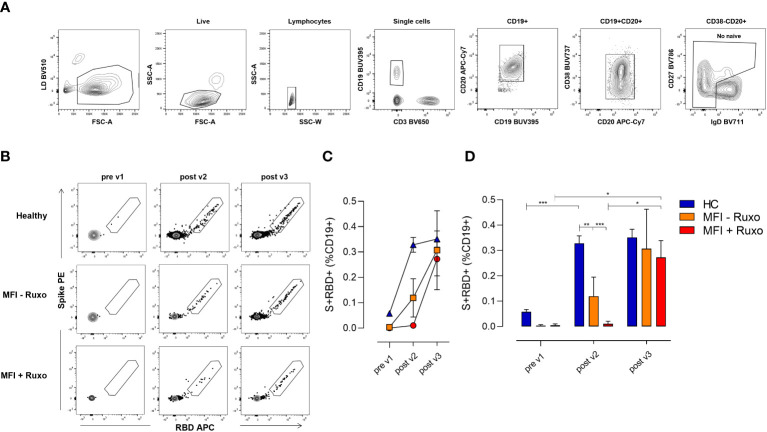
Spike-specific B cell response following mRNA SARS-CoV-2 vaccination in patients with myelofibrosis (MF), with or without ruxolitinib (Ruxo) treatment, after second and third vaccine dose. Identification of spike (S) and RBD-specific B cells by flow cytometry within PBMCs collected at the baseline (pre v1), after two (post v2) and three (post v3) doses of mRNA SARS-CoV-2 vaccine. **(A)** Gating strategy for identifying CD19+ spike/RBD-specific B cells (named S+RBD+ B cells) by multiparametric flow cytometry. **(B)** Representative dot plot analysis of spike PE *versus* RBD APC within CD19+ cells, for identifying S+RBD+ B cells in MF subjects with and without therapy, and in healthy controls (HC). **(C)** Mean value (±SEM) of S+RBD+ B cells percentages at different time points evaluated in MF subjects with and without therapy, and in HC. **(D)** Frequencies of S+RBD+ B cells on CD19+ cells at different time points evaluated in MF subjects with and without therapy, and in HC. Kruskal-Wallis test, followed by Dunn’s post test for multiple comparisons, was used for assessing statistical differences between different groups each time point. Unpaired Mann-Whitney test was used to assess statistical difference at each group at different time points. *P ≤ 0.05; **P ≤ 0.01; ***P ≤ 0.001.

The induction of the B cell response upon vaccination is characterized by the production of B cell subpopulations with different functionalities and phenotypes, according to the effector or memory phase of the immune response. Here, we characterized the spike-specific B cell response at the baseline (pre v1), 7 days after the second (post v2) and 15 days after the third (post v3) vaccination in MF patients and HC. SARS-CoV-2 specific B cells were identified using the full-spike protein and RBD as fluorescent probes, and their phenotype was characterized assessing the expression of CD19, CD20, IgD, CD27, CD38, molecules. Spike/RBD-specific cells were detected within the pool of not naïve CD19^+^ cells (hereafter named S^+^RBD^+^ B cells, **Figures 3A, B**).

S^+^RBD^+^ B cells were elicited in blood of MF patients without ruxolitinib treatment after two vaccine doses (post v2) as shown in **Figures 3C, D**, with a significantly lower percentage compared to HC group (frequency of 0.13% and 0.32%, respectively; P≤ 0.01). Undetectable SARS-CoV-2 specific B cells were instead observed in patients treated with ruxolitinib (0.01%). The third dose significantly boosted the SARS-CoV-2 specific B cell response in MF patients with an increase of S^+^RBD^+^ B cells to 0.30% and 0.28% in patients without or with ruxolitinib treatment, respectively (P≤0.05), stimulating a B cell response similar to that observed in HC (**Figures 3C, D**). The analysis of the spike^+^RBD^+^specific memory B cell response strongly highlights the crucial impact of the third dose on the improvement of the response to the COVID-19 vaccination in this cohort of fragile patients.

### Analysis of antibody and B cell response in individual MF patients

**Figure 4:**
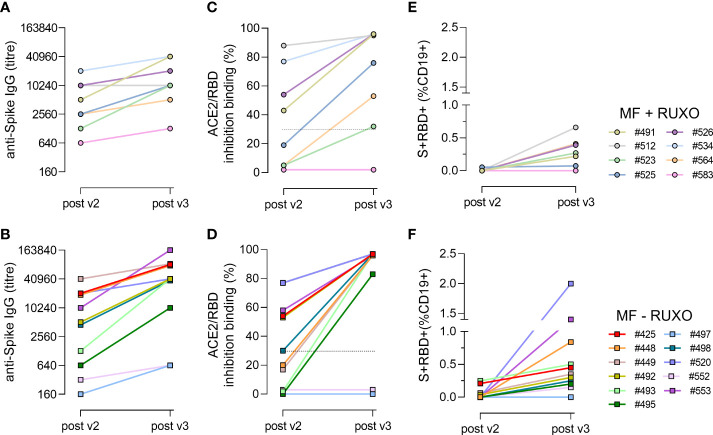
Time course of spike-specific antibody and B cell responses in plasma of myelofibrosis (MF) patients after the second and third SARS-CoV-2 mRNA vaccine dose. Nineteen MF patients, 8 with ruxolitinib (Ruxo) treatment **(A, C, E)** and 11 without therapy **(B, D, F)**, were monitored after two (post v2) and three (post v3) doses of mRNA SARS-CoV-2 vaccine to study the kinetic of spike-specific IgG **(A, B)**, the ACE2/RBD binding inhibition activity **(C, D)** and the spike/^+^RBD^+^-specific B cells generated **(E, F)**. Each subject is identified with a number and a color, as reported in the legend.

The longitudinal profiling of the humoral and B cell responses in each patient following the second and third vaccine dose, subdividing patients with (n=8) or without (n=11) ruxolitinib treatment, is reported in **Figure 4**, while clinical parameters of each subject are summarized in **Table S1**.

The time course analysis of anti-spike IgG clearly shows a two-fold increase in each individual following the third dose, except for patient #583 under ruxolitinib treatment (**Figure 4A**) and for patients #497 and #552 without ruxolitinib treatment (**Figure 4B**), which maintained a low antibody response also after the booster dose. Concomitantly, the three patients were negative for the ACE2/RBD inhibition binding activity (**Figures 4C, D**) and did not develop a spike-specific B cell response (**Figures 4E, F**). Patient #583 was on ruxolitinib and ibrutinib treatment, as he is affected by chronic lymphocytic leukemia as well. His lower immune responsiveness to SARS-CoV-2 vaccination can therefore be due to the immunosuppressive ibrutinib drug, that inhibits B-cell proliferation and survival. Patients #497 and #552, who received only HU or supportive therapy (**Table S1**), have been treated with, or they were still receiving lymphodepleting therapies at the time of vaccination. In fact, patient #552 was treated for a non-Hodgkin lymphoma few years before, receiving six cycles of immunochemotherapy (R-COP) and a rituximab maintenance therapy, while patient #497 was on chronic therapy with methylprednisolone for fibromyalgia.

In all the other patients, the third dose of mRNA vaccine considerably enhanced SARS-CoV-2-specific antibody and B-cell responses, also in low responder subjects after the second dose (#523, #564, #525), independently of the ruxolitinib treatment. S^+^RBD^+^ B cells were observed in 7/8 (87%) ruxolitinib-treated and 7/11 (64%) untreated patients (**Figures 4E, F**).

## Discussion

Booster doses of COVID-19 vaccines have been strongly recommended for fragile subjects by regulatory authorities. In the present work we demonstrated the impact of the third dose with an mRNA-based vaccine on the immune responsiveness of MF patients, with or without ruxolitinib treatment. To deeply dissect the immune mechanisms elicited by the third dose, we analyzed the amount and the functionality of spike-specific antibodies and the generation of the spike-specific memory B cells, that play a crucial role in response to pathogen encounter.

We previously demonstrated a slower kinetics of antibody response in MF patients at early time points after the second dose of mRNA SARS-CoV-2 vaccine compared to healthy subjects, and a reduced ACE2/RBD inhibition binding especially in subjects under ruxolitinib treatment.

The analysis of the antibody response following the first vaccination cycle in healthy and fragile subjects (21, 24–26) indicates that factors such as age, sex, and comorbidities have shown impact on the level of spike-specific IgG levels and the rate at which they decline overtime (27, 28). Age and comorbidities, which can induce physiological and premature immune senescence, respectively, as well as chronic systemic low-grade inflammation have been considered among the most impacting factors on immune responsiveness to vaccination (29–31). Applying a linear multivariate model to assess the impact of the age and sex as confounding factors on the antibody response in this study, no significant effect was observed (data not shown). This can be due to the low number of subjects and a skewing towards an older age of the patients (68% was > 70 years), which constitute a limitation to the present work.

The reduced capability of the immune system of MF patients to promptly react to vaccination, strongly suggested the need of a third vaccine dose (11). Public health agencies have advised the administration of a booster dose 4-6 months after the primary series of vaccination is completed, to strengthen protection against serious illness and death from COVID-19, especially for fragile patients. Several studies have described the effect of the third vaccine dose on the humoral immune response and on the protection from infections, without relevant adverse events (32–36). We recently provided evidence that a third dose of mRNA-1273 vaccine, in subjects primed with two doses of the same vaccine, substantially increase SARS-CoV-2-specific antibody and B-cell response in low responder hematopoietic cell transplantation recipients, emphasizing the importance of an additional vaccine dose for those who may have produced a low response upon the primary series of the COVID-19 vaccination (13). The administration of the third dose has opened the door to heterologous prime-boost COVID-19 vaccination approaches, obtained by the combination of vector- and mRNA-based vaccines, or mixed mRNA platforms (37, 38). Here, the cohort of MF patients included 17 subjects vaccinated with homologous mRNA vaccines and only 2 primed with Spikevax and boosted with Comirnaty mRNA vaccine, therefore it was not possible to make a comparison of immunogenicity between the different prime-boost strategies. Nevertheless, data obtained in healthy subjects support the heterologous combination as a successful strategy for improving immunogenicity and safety, as well as a practical solution to shortages of some vaccines, or to changes in national authorization for some initially licensed vaccines (39, 40) (Pastore et al, in preparation). This approach should be taken in consideration also for vaccination strategies tailored for fragile patients.

Here, we demonstrated the strong booster effect of the third dose in MF patients in terms of spike-specific IgG levels, ACE2/RBD inhibition binding ability and spike-specific B cells. While other studies have investigated the effect of the third dose only on the antibody response, here we profiled the memory B cells specific for SARS-CoV-2. The characterization of the spike-specific B cell response is particularly relevant since the ability of memory B cells to be promptly re-activated by pathogen encounter and differentiate into antibody-secreting plasmacells, capable of secreting virus neutralizating antibodies (19). This booster effect was particularly relevant for MF patients that were low responder after the second vaccine dose, independently from ruxolitinib treatment, as reported also by Caocci et al. in MF patients vaccinated with BNT162b2 mRNA COVID-19 vaccine (15). On the other hand, Auteri at al demonstrated an impaired early humoral response to the SARS-CoV-2 vaccine in Philadelphia-negative chronic myeloproliferative neoplasms patients receiving ruxolitinib (41), and extended this issue to the later response as well (14). The speculation about the impact of ruxolitinib treatment and of the exposition time on the immune response needs to be further investigated, considering the small number of subjects included in difference studies.

Moreover, we observed a similar frequencies of spike-specific B cells between MF, treated or untreated with ruxolitinib therapy, and HC after the third dose, while a significant lower response in MF was detected after two vaccine doses. These data, showing the slower evolution of spike-specific B cells in MF after two doses, corroborate our previous data on the different kinetic of spike-specific antibody response in MF patients compared to HC, and suggest a reduced capability of their immune system to be rapidly boosted (11). Despite the observed positive impact of the third dose in most of the MF patients, we observed and reported the case of three patients that maintained a low response also after the booster dose. One of three low responder patients was receiving ruxolitinib treatment at the same time of the first vaccine dose, while the other two were under HU/supportive therapies. Interestingly, all the three patients had received or were receiving lymphodepleting therapies for other pathologies. Patient #583, affected also by chronic lymphocytic leukemia, is in treatment with ibrutinib; patient #552 received a B-cell-depleting treatment with anti CD20 monoclonal antibodies for a non-Hodgkin lymphoma some years ago, without IgG recovery before the vaccination, and patient #497, affected by fibromyalgia, is in chronic therapy with methylprednisolone, that induces lymphopenia and hypogammaglobulinemia. As such, in line with other recent reports, the lower response to mRNA vaccines in hematologic patients seems to be influenced by B-cell depleting therapies, hypogammaglobulinemia (42) and immunosuppressive treatment (43), regardless of therapy with ruxolitinib.

These results contribute to answer the open question on the magnitude of spike-specific antibody and B-cell response elicited by COVID-19 mRNA vaccines in MF patients, and on the impact of ruxolitinib treatment on the humoral and cellular immune response generated. Our findings demonstrate that a third dose of mRNA vaccine considerably enhances spike-specific antibody and B-cell response in MF patients, regardless of therapy with ruxolitinib. Data highlight the importance of the third vaccine dose to strongly boost immune response for those who may have achieved a limited response to the primary cycle of COVID-19 vaccination. This knowledge is crucial to assess the need for additional booster doses of SARS-CoV-2 mRNA vaccines and to guide vaccination policies designed for MF patients.

## Data availability statement

The raw data supporting the conclusions of this article will be made available by the authors, without undue reservation.

## Ethics statement

The study was performed in compliance with all relevant ethical regulations and the protocol was approved by local Ethical Committee for Clinical experimentation of Regione Toscana Area Vasta Sud Est (CEASVE), protocol code 19479 PATOVAC COV v1.0, approved on March 15^th^ 2021, and protocol code 18869 IMMUNO_COV v1.0, approved on November 18^th^ 2020. The patients/participants provided their written informed consent to participate in this study.

## Author contributions

AC, GP, FM, ASi, MB, DM conceived the study. GP, ASa, MS, SG, CB, VS, FM, VS, MB. enrolled patients. FF, GP, JP processed the samples. FF, AC, GP, JP carried out the immunological analysis. FF, AC, GP, JP, DM. analyzed the data. FF, AC, MB, DM wrote the manuscript. FF, AC, GP, MB, DM. supervised the study. DM provided financial support. All authors contributed to the article and approved the submitted version.

## Funding

This study was supported by the Department of Medical Biotechnologies of the University of Siena.

## Acknowledgments

We would like to thank all the volunteers who participated in the study and the Hematology Unit nursing staff, who chose to cooperate for blood withdrawal. We thank Simone Lucchesi for the helpful discussion concerning data analysis.

## Conflict of interest

The authors declare that the research was conducted in the absence of any commercial or financial relationships that could be construed as a potential conflict of interest.

## Publisher’s note

All claims expressed in this article are solely those of the authors and do not necessarily represent those of their affiliated organizations, or those of the publisher, the editors and the reviewers. Any product that may be evaluated in this article, or claim that may be made by its manufacturer, is not guaranteed or endorsed by the publisher.
