# Supplementary material for: The third dose of mRNA SARS-CoV-2 vaccines enhances the spike-specific antibody and memory B cell response in myelofibrosis patients
Source: Front Immunol. 2022 Sep 29;13:1017863. doi: 10.3389/fimmu.2022.1017863 (PMC9556722; doi:10.3389/fimmu.2022.1017863)
Supplement: Supplementary file 1 [file Table_1.pdf]

The third dose of mRNA SARS-CoV-2 vaccines enhances the spike-specific antibody and memory B cell response in myelofibrosis patients

Fabio Fiorino, Annalisa Ciabattini, Anna Sicuranza, Gabiria Pastore, Adele Santoni, Martina Simoncelli, Jacopo Polvere, Sara Galimberti, Claudia Baratè, Vincenzo Sammartano, Francesca Montagnani, Monica Bocchia, Donata Medaglini**

Table S1. Clinical characteristics and treatment of each patient that received the third vaccine dose of mRNA vaccine anti SARS-CoV-2. Patients under treatment with ruxolitinib are reported in the upper part of the table, the ones without treatment are reported below. Patients were listed in descending order of anti-spike antibody response. The three non-responders are reported in bold.

	Age	Sex	Disease	IPSS score	Driver mutation	Therapy	Spleen* (cm)	Hb (g/dl)	WBC $x10^3$/ µL	PLT $x10^3$/µL	Ly $x10^3$/ µL	LDH (U/L)	Total proteins (g/dl)	γ-globulins (%)
PAT#534	75	M	PMF	4	JAK2	RUXO	5	13.4	29.6	149	3.55	838	7.9	23,3
PAT#491	80	F	PMF	1	JAK2	RUXO	22	11.7	3.67	27	1.34	786	6.4	14,1
PAT#526	64	F	PPV	1	JAK2	RUXO	12.6	9.5	4.39	217	1.07	334	NA	NA
PAT#523	47	F	PPV	0	JAK2	RUXO	13	12.7	5.30	565	1.40	235	NA	NA
PAT#525	65	F	PPV	1	JAK2	RUXO	12	12.4	6.94	680	1.48	422	NA	NA
PAT#512	88	F	PPV	2	JAK2	RUXO	0	10.4	13.13	532	2.30	361	NA	NA
PAT#564	72	M	PTE	1	JAK2	RUXO	12	12.4	33.94	134	1.35	583	NA	NA
**PAT#583**	**85**	**F**	**PMF**	**3**	**JAK2**	**RUXO**	**14**	**13**	**33.47**	**70**	**6.02**	**315**	**6**	**15,2**
PAT#553	77	M	PTE	1	CALR	HU	5	11.1	5.01	621	1.43	555	6.9	15,5
PAT#449	83	F	PMF	2	None	HU	5	8.6	11.50	60	3.79	443	6.9	19,9
PAT#425	44	F	PMF	0	CALR	HU	0	13.1	5.15	618	1.80	221	NA	NA
PAT#448	69	M	PMF	2	CALR	HU	8	10.8	5.87	253	1.76	1384	6.6	14
PAT#520	78	M	PMF	1	CALR	NONE	0	12.7	10.30	601	2.40	191	7.1	13,6
PAT#498	69	F	PPV	0	JAK2	HU	1	14.8	9.78	395	2.80	301	NA	NA
PAT#492	79	M	PMF	1	JAK2	HU	0	14.4	7.03	262	2.13	215	6.7	19,1
PAT#493	63	F	PMF	0	CALR	HU	0	12.2	4.30	781	2.02	326	7.1	15,2
PAT#495	71	M	PMF	3	JAK2	HU	13	10.6	13.76	141	1.23	666	6.1	13,5
**PAT#552**	**78**	**M**	**PMF**	**2**	**JAK2**	**HU**	**8**	**14.2**	**11.64**	**760**	**0.93**	**469**	**5.7**	**7,3**
**PAT#497**	**82**	**F**	**PMF**	**1**	**JAK2**	**HU**	**9**	**10.6**	**9.45**	**458**	**1.32**	**562**	**NA**	**NA**

PMF=primary myelofibrosis; PPV-MF=post-polycythemia vera myelofibrosis; PET-MF=post-essential thrombocythemia myelofibrosis; RUXO=ruxolitinib; HU=hydroxyurea; Hb=hemoglobin (g/dl); WBC=white blood count (x103/ µL); PLT=platelet count (x103/ µL); Ly=lymphocytes (x103/ µL); LDH=lactate dehydrogenase (U/L). *Spleen below costal margin
